# *TREM2* splice isoforms generate soluble TREM2 species that disrupt long-term potentiation

**DOI:** 10.1186/s13073-023-01160-z

**Published:** 2023-02-20

**Authors:** Miguel Moutinho, Israel Coronel, Andy P. Tsai, Gonzalo Viana Di Prisco, Taylor Pennington, Brady K. Atwood, Shweta S. Puntambekar, Daniel C. Smith, Pablo Martinez, Seonggyun Han, Younghee Lee, Cristian A. Lasagna-Reeves, Bruce T. Lamb, Stephanie J. Bissel, Kwangsik Nho, Gary E. Landreth

**Affiliations:** 1 Stark Neurosciences Research Institute, Indiana University School of Medicine, Indianapolis, IN USA; 2 Department of Anatomy, Cell Biology and Physiology, Indiana University School of Medicine, Indianapolis, IN 46202 USA; 3 Department of Pharmacology and Toxicology, Indiana University, School of Medicine, Indianapolis, IN 46202 USA; 4 Department of Medical and Molecular Genetics, Indiana University School of Medicine, Indianapolis, IN USA; 5 Department of Biomedical Informatics, University of Utah School of Medicine, Salt Lake City, UT USA; 6 Department of Radiology and Imaging Sciences, Indiana Alzheimer’s Disease Research Center, Indiana University School of Medicine, Indianapolis, IN USA

**Keywords:** TREM2, Soluble TREM2, TREM2 splicing, Alzheimer’s disease

## Abstract

**Background:**

TREM2 is a transmembrane receptor expressed by myeloid cells and acts to regulate their immune response. TREM2 governs the response of microglia to amyloid and tau pathologies in the Alzheimer’s disease (AD) brain. TREM2 is also present in a soluble form (sTREM2), and its CSF levels fluctuate as a function of AD progression. Analysis of stroke and AD mouse models revealed that sTREM2 proteins bind to neurons, which suggests sTREM2 may act in a non-cell autonomous manner to influence neuronal function. sTREM2 arises from the proteolytic cleavage of the membrane-associated receptor. However, alternatively spliced TREM2 species lacking a transmembrane domain have been postulated to contribute to the pool of sTREM2. Thus, both the source of sTREM2 species and its actions in the brain remain unclear.

**Methods:**

The expression of TREM2 isoforms in the AD brain was assessed through the analysis of the Accelerating Medicines Partnership for Alzheimer’s Disease Consortium transcriptomics data, as well as qPCR analysis using post-mortem samples of AD patients and of the AD mouse model 5xFAD. TREM2 cleavage and secretion were studied in vitro using HEK-293T and HMC3 cell lines. Synaptic plasticity, as evaluated by induction of LTP in hippocampal brain slices, was employed as a measure of sTREM2 actions.

**Results:**

Three distinct TREM2 transcripts, namely ENST00000373113 (TREM2^230^), which encodes the full-length transmembrane receptor, and the alternatively spliced isoforms ENST00000373122 (TREM2^222^) and ENST00000338469 (TREM2^219^), are moderately increased in specific brain regions of patients with AD. We provide experimental evidence that TREM2 alternatively spliced isoforms are translated and secreted as sTREM2. Furthermore, our functional analysis reveals that all sTREM2 species inhibit LTP induction, and this effect is abolished by the GABAA receptor antagonist picrotoxin.

**Conclusions:**

TREM2 transcripts can give rise to a heterogeneous pool of sTREM2 which acts to inhibit LTP. These results provide novel insight into the generation, regulation, and function of sTREM2 which fits into the complex biology of TREM2 and its role in human health and disease. Given that sTREM2 levels are linked to AD pathogenesis and progression, our finding that sTREM2 species interfere with LTP furthers our understanding about the role of TREM2 in AD.

**Supplementary Information:**

The online version contains supplementary material available at 10.1186/s13073-023-01160-z.

## Background

Alzheimer’s disease (AD) is the most common form of dementia for which there is no effective treatment. The AD brain is typified by a robust microglial immune response triggered by Aβ accumulation, accompanied by progressive neurodegeneration and cognitive decline [1, 2]. Genetic studies have linked many immune genes subserving this microglial response to the risk of late-onset AD (LOAD) [3, 4]. The triggering receptor expressed in myeloid cell 2 (TREM2) is among these immune genes, as its genetic variants, such as R47H or H157Y, confer a significantly increased risk of LOAD [5–8]. TREM2 encodes a single pass membrane receptor that employs the signaling adapter DNAX activation protein 12 (DAP12 or TYROBP) to transduce signals elicited by binding of its ligand(s). In the brain, TREM2 expression is restricted to microglia where it acts to promote microglial survival and proliferation, as well as it stimulates microglial engagement and uptake of amyloid plaques in AD models [9, 10].

In the brain, TREM2 is present as a full-length membrane receptor or as a soluble protein (sTREM2). sTREM2 is generated from proteolytic cleavage of membrane-bound TREM2 receptor at residue H157 by ADAM10, ADAM17, or other unidentified proteinases, releasing the extracellular N-terminal fragment [11, 12]. Interestingly, alternative splicing of *TREM2* generates two splice isoforms that lack the transmembrane domain and have been postulated to contribute to the pool of sTREM2 [13–15]. The transcript ENST00000373113 (TREM2^230^) encodes for the 230 amino acid (aa) full-length transmembrane receptor, and the spliced isoforms lacking the transmembrane domain are encoded by ENST00000373122 (TREM2^222^) and ENST00000338469 (TREM2^219^), respectively. Although these alternatively spliced transcripts exist in the human brain [13, 16–18], it has not been established whether they can be translated to generate sTREM2 species. TREM2^230^, TREM2^222^, and TREM2^219^ share an identical N-terminal sequence but have distinct C-termini [15].

The potential of sTREM2 as a biomarker for AD has been widely studied. Increasing evidence supports that CSF sTREM2 levels fluctuate as a function of different stages of AD, with higher concentrations of sTREM2 correlated with slower disease progression [19–25]. These studies have employed immunoassays that target the TREM2 N-terminus, which does not differentiate between the TREM2^230^ fragment cleaved at H157 (sTREM2^H157^) and the other splice isoforms since they all share an identical N-terminal sequence. Nonetheless, a recent large longitudinal analysis of the dominantly inherited Alzheimer network (DIAN) used a novel assay with an antibody that specifically detects sTREM2^H^^157^, but not other splice isoforms in human CSF [26]. This study revealed that an augmented rate of increase in CSF sTREM2^H^^157^ during the symptomatic stage of the disease correlates with a decreased rate of amyloid deposition. Interestingly, an enhanced longitudinal rate of increase in CSF sTREM2^H^^157^ during the presymptomatic phase correlated with decreased cortical shrinkage and cognitive decline.

sTREM2 has been shown to directly bind to neurons in mouse models of stroke and AD [27, 28], which suggests that sTREM2 exerts cell non-autonomous actions on neuronal function. The expression of specific *TREM2* transcripts in the brain tissue of AD patients has been previously analyzed [16, 17, 29]; however, these studies did not yield consistent results, and it remains unclear whether the levels of *TREM2* transcripts change in AD. Interestingly, *TREM2*^230^, *TREM2*^222^, and *TREM2*^219^ expression was found to be increased in the brain tissue of patients with progressive supranuclear palsy (PSP) [18]. These authors observed a positive correlation between TREM2^230^ and TREM2^219^ expression with hyperphosphorylated tau burden in neurons. Moreover, recent studies have reported that sTREM2 activates microglia and rescues long-term potentiation (LTP) in the 5xFAD mouse model of AD [30, 31]; however, the levels of sTREM2 used in these studies (20–160 nM ≈ 380–3000 ng/ml) are dramatically higher than the concentration reported for sTREM2 in the CSF of patients with AD, which is usually found on the low ng/ml range (approximately between 0.1–25 ng/ml) [19–25, 32]. Although these studies support a link between neuronal function and sTREM2, the endogenous role of these proteins remains unclear.

Herein, we show that TREM2 splice transcripts increase in different regions of the AD brain. Importantly, we provide robust evidence that different splice isoforms are translated and produce a heterogeneous pool of sTREM2 species. Furthermore, we report that these sTREM2 species interfere with neuronal function by inhibiting LTP. These novel insights into the generation and function of sTREM2 are important to further our understanding about the complex biology of TREM2 in health and disease.

## Methods

### *TREM2* expression analysis using AMP-AD datasets

The generation of RNA-Seq data in the Accelerating Medicines Partnership for Alzheimer’s Disease Consortium (AMP-AD) and demographic information has been previously described in detail [33]. Briefly, RNA-Seq data were downloaded from the (AMP-AD) through the Synapse database (https://www.synapse.org/): the Mayo Clinic Brain Bank (Mayo Clinic) [34], the Mount Sinai Medical Center Brain Bank (MSBB) [35], and the Religious Orders Study and Memory and Aging Project (ROS/MAP) cohorts [36].

In the Mayo Clinic, RNA-Seq data were generated from the temporal cortex and cerebellum. In the MSBB, RNA-Seq data were generated from the parahippocampal gyrus, inferior frontal gyrus, superior temporal gyrus, and frontal pole. In ROSMAP, RNA-Seq data were generated from the dorsolateral prefrontal cortices. The procedures for sample collection, post-mortem sample descriptions, tissue and RNA preparation methods, library preparation and sequencing methods, and sample quality controls were previously described in detail [34–39]. We converted each mapped BAM file into a FASTQ file using samtools v.1.9 and then re-mapped the converted FASTQ files onto the hg19 human reference genome using STAR aligner v.2.5, as previously described in detail [40]. Using the processed RNA-Seq data, we identified *TREM2* splice transcripts and calculated their expression levels. We used the software tool RSEM to accurately estimate the *TREM2* transcripts expressions from RNA-Seq [41]. RSEM generates three different *TREM2* transcript sequence references, and RNA-Seq reads are mapped to them. After the alignment of reads, RSEM uses a statistical model to accurately calculate transcript abundances by estimating a maximum likelihood (ML) based on expectation-maximization (EM) algorithm. Additionally, by utilizing paired-end reads to classify the different isoforms, RSEM improves the estimation of the relative isoform levels within single genes. Based on RSEM’s statistical model and additional benefits, it accurately estimates transcript abundances from reads mapped to distinct and shared regions among the three isoforms. Differential expression analysis of the *TREM2* splice transcripts between cognitively normal controls and AD patients was done using a generalized linear regression model [33]. The regression was performed with the “glm” function of the stats package in R (version 3.6.1). Age and sex were used as covariates. We used the false discovery rate (FDR) to correct for multiple testing.

### Mouse models

5xFAD mice were obtained from the Jackson Laboratory (Stock #34840-JAX) and express five human familial Alzheimer’s disease mutations driven by the mouse Thy1 promoter (APPSwFlLon, PSEN1*M146L*L286V]6799Vas) [42]. The APPPS1–21 mouse model expresses the Swedish APP mutation (KM670/671NL) and the L166P mutation in PSEN1 driven under the Thy-1 promoter [43]. Cortical RNA from the transgenic mouse model that harbors the human *TREM2* BAC (control B6^hTREM2^ and 5xFAD^hTREM2^) [35] was kindly provided by Dr. Daniel Lee and Dr. William Yang (Semel Institute, UCLA). To collect brain tissue, mice were anesthetized with 1.2% 2,2,2-tribromoethanol (Avertin), perfused with ice-cold phosphate-buffered saline (PBS), and the brain removed. Brain tissue was used for RNA extraction. All animals were maintained, and experiments were performed in accordance with the recommendations in the Guide for the Care and Use of Laboratory Animals of the National Institutes of Health. The protocol was approved by the Institutional Animal Care and Use Committee (IACUC) at Indiana University School of Medicine.

### Human samples

Postmortem brain tissue (middle frontal gyrus) from control and AD subjects were provided in the form of frozen blocks by the Brain Resource Center at Johns Hopkins. AD samples were examined at the Division of Neuropathology at John Hopkins University and consisted of pathologically severe AD, stages V–VI (Additional file 1: Table S1). The brains were kept at − 80 °C until used.

### RNA extraction and quantitative real-time PCR

**Fig. 1:**
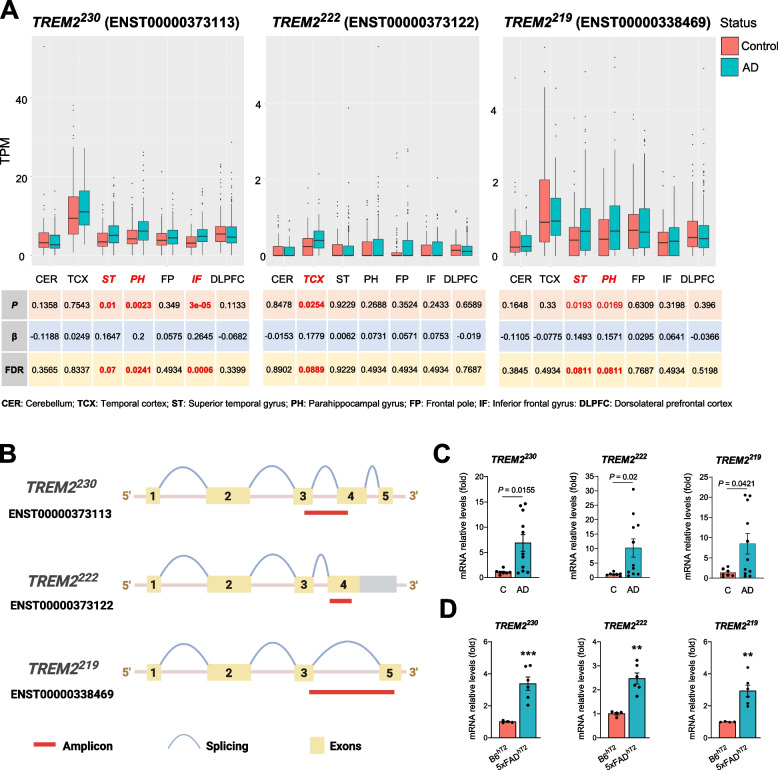
*TREM2* isoforms expression increases in Alzheimer’s disease. **A** Expression analysis of *TREM2* isoforms (*TREM2*^230^, *TREM2*^222^, and *TREM2*^219^) in different brain regions in control subjects (Control) and patients with late-onset Alzheimer’s disease (AD) using the AMP-AD dataset. **B** Design of qPCR primers to specifically detect *TREM2* isoforms. **C** qPCR expression analysis of *TREM2* isoforms in middle frontal gyrus tissue of control subjects (C) and patients with Alzheimer’s disease (AD). Statistical analysis was performed by the Student *t*-test for *TREM2*^230^ and Mann-Whitney test for *TREM2*^222^ and *TREM2*^219^. **D** qPCR expression analysis of *TREM2* isoforms in brain cortical tissue of 7-month-old control and 5xFAD mice expressing human *TREM2* gene (B6^hT2^and 5xFAD^hT2^). Animals from both sexes were analyzed together (3 males and 1 female B6^hT2^; 3 males and 3 females 5xFAD^hT2^). Statistical analysis was performed by the Student *t*-test. Data are expressed as mean values ± SEM (**P < 0.01; ***P < 0.001)

Homogenates from 20 mg of human postmortem tissue were used for RNA isolation (Qiagen #74104); 200 ng of RNA was converted to cDNA with the High-Capacity cDNA Reverse Transcription Kit (Applied Biosystems 4368814). Murine tissue was homogenized in buffer containing 1% NP-40, 0.5% sodium deoxycholate, 0.1% SDS, and protease inhibitor cocktail (Sigma Aldrich, P8340) and mixed in an equal volume of RNA-Bee (Amsbio, CS-104B). RNA was isolated using phenol-chloroform extraction and a Purelink RNA Mini Kit (Life Technologies, 12183020) with an on-column DNAse Purelink Kit (Life Technologies, 12183025); 500–1000 ng of RNA was converted to cDNA with the High Capacity RNA-to-cDNA kit (Applied Biosystems, 4388950). qPCR was performed on StepOne Real-Time PCR System with PowerUp™ SYBR™ (Thermo Fisher Scientific A25742). The mRNA levels of mouse *Trem2* and human *TREM2* were normalized to *Gapdh* and *GAPDH*, respectively, and expressed as fold changes relative to controls, using the ΔΔCt method. Both mouse and human *TREM2* transcripts sequences were retrieved from NCBI, and specific nucleotide sequences for each transcript were identified by analyzing the sites of splicing events (exon skipping, frameshift, and sequence insertions). These unique sequences were used to design the primers that amplify specific amplicons for each transcript (Fig. 1B). The primer sequences are available in Additional file 1: Table S2. The qPCR reactions were performed in a StepOnePlus™ Real-Time PCR (Applied Biosystems) with 40 amplification cycles with each cycle consisting of 95°C for 15 s followed by 1 min at 60 °C for all isoforms, except for the human *TREM2*^230^, for which we used a temperature of 68 °C.

### Plasmid design, cell culture, and transfection

Custom TREM2 plasmids were generated by GeneArt Gene Synthesis (Thermo Fisher Scientific) in the pcDNA3.1(+) backbone. The plasmids encoded for human and mouse TREM2 isoforms harboring a N-terminus HA tag and a C-terminus FLAG tag connected with short linker sequences (Additional file 1: Table S3). The human embryonic kidney 293T (HEK-293T) and human microglia cell line (HMC3) were cultured and maintained in Dulbecco’s modified Eagle’s medium (DMEM) high glucose, with GlutaMAX™ (Gibco, 10566024), supplemented with 10% fetal bovine serum (FBS) (Gibco, 16000) and 1% penicillin-streptomycin (Gibco, 15140). TREM2 plasmids were transfected into both cell types using Lipofectamine 3000 (Thermo Fisher Scientific, L3000015) according to the manufacturer’s protocol. HMC3 cells were transfected for 24 h and used for immunocytochemistry. HEK-293T transfected for 24 h were used for RNA extraction using the DNAse Purelink Kit, followed by cDNA conversion and qPCR as described above. HEK-293T transfected for 48 h were maintained with media without FBS after transfection, and protein extracts were collected from cells and media.

### Immunocytochemistry

Cells were washed with PBS and fixed with paraformaldehyde 4% in PBS for 15 min at room temperature. Subsequentially, cells were washed with PBS and incubated with blocking buffer (5% BSA, 0.1% Triton, and 0.1% Tween-20) for 1 h at room temperature, followed by overnight incubation with primary antibody for HA 1:500 in blocking buffer (Cell signaling, C29F4) at 4 °C. Afterwards, cells were washed with PBS and incubated with secondary antibody (Alexa Fluor™ donkey anti-rabbit) 1:1000 and DAPI (1 μg/ml) in blocking buffer for 1 h at room temperature. Cells were washed with Tween 0.01% in PBS and mounted with ProLong™ Gold Antifade Mountant (Thermo P36930). Images were taken using the Nikon AR1 confocal microscope.

### Protein extraction

Cells were lysed using a lysis buffer containing 1% Triton-X 100, 50 mM Tris-HCl pH = 8, and 150 mM NaCl, sonicated followed by centrifugation at 12.000*g* for 15 min at 4 °C. Human brain tissue was mechanically homogenized with a glass tissue homogenizer (Dounce) in Tris-buffered solution (TBS—150 mM NaCl, 50 mM Tris-HCl, pH 7.6) or RIPA (50 mM TrisHCl, pH 7.4, 150 mM NaCl, 1% Triton X-100, 0.5% sodium deoxycholate, 0.1% SDS, 1 mM EDTA), followed by sonication and centrifugation for 10 min, and the supernatant (soluble fraction) was collected. All lysis buffers were supplemented with protease and phosphatase inhibitors. Protein concentration was determined with a BCA kit (Thermo Scientific).

We used trichloroacetic acid (TCA) to precipitate proteins from transfected HEK-293T-conditioned media without FBS. Conditioned media was filtered through a 0.2-μm pore size filter and mixed with TCA 20% in the ratio of 50:50 (volume), followed by incubation on ice for 1 h. After that, samples were centrifuged at 4 °C for 5 min at 10.000*g*, and the supernatant was discarded. The pellets were washed with ice-cold acetone followed by centrifugation at 4 °C for 5 min at 10,000*g*. The washing step was performed 3 times, the pellet was dried at room temperature, and then resuspended in the western blot loading buffer.

### Western blot

Protein extracts were heated for 5 min at 95 °C, loaded into 4–12% Bis-Tris gels (Life Technologies), and run at 150V. Proteins were transferred into immobilon-P PVDF membranes at 400 mA, blocked in 5% milk in TBS-Tween 0.1% (TBST), and incubated with primary antibodies overnight at 4 °C. All secondary HRP-conjugated antibodies were incubated for 1 h at room temperature. The following primary antibodies were used: Anti-HA-Tag (Cell Signaling, C29F4), Anti-FLAG® (Sigma-Aldrich, F1804), Anti-DYKDDDDK Tag (Cell Signaling, D6W5B), anti-β-actin (Santa Cruz, sc-47778), anti-α-tubulin (Licor, 926-42213), anti-vinculin (Sigma-Aldrich, V9131), anti-hTREM2 (R&D AF1828), anti-TREM2^222^ (Ab222), and anti-TREM2^219^ (Ab219). Ab222 and Ab219 are custom monospecific antibodies generated by Pacific Immunology. The antigen for Ab222 is CSLAWTEARDTSTQ, and for Ab219 is RAERHVKEDDGRKSPGEVPPGTS-Cys. Rabbits were immunized, and their serum was used to purify antibodies by affinity purification against the above mentioned protein sequences. This process allows the isolation of highly specific antibodies that approach the specificity of monoclonal antibodies and provide the superior affinity of polyclonal antibodies (https://www.pacificimmunology.com). All antibodies were diluted in 5% milk in TBST, except for anti-vinculin which was dissolved in 5% bovine serum albumin (BSA) in TBST.

### sTREM2 production, purification, and quantification

HEK-293T cells were transfected with TREM2 plasmids as described above and maintained in FBS-free media for 48 h. Conditioned media were collected, filtered with a 0.2-μm pore size filter, and concentrated using Pierce Protein Concentrators (Thermo Fisher Scientific, 88535). Afterwards, we purified sTREM2 from concentrated conditioned media using the HA-tagged Protein Purification Kit (MBL International, 3320) and following the manufacturer’s guidelines. This kit allows HA-tagged protein to be purified based on the use of HA beads and competitive elution. Proteins were stored in PBS and glycerol (50:50) at − 20 °C. Soluble TREM2 species were visualized by silver staining using the Pierce™ Silver Stain Kit (Thermo Fisher Scientific, 24612) after electrophoresis in 4–12% Bis-Tris gel in denaturing and reducing conditions. Soluble TREM2 proteins were quantified by ELISA, similar to what has been previously described [44]. Briefly, F8 Maxisorp Nunc-Immuno Module (Thermo Fisher, 468667) wells were coated with 2 μg/ml of the TREM2 capture antibody (R&D Systems, MAB17291) in 0.05 M carbonate/bicarbonate buffer (pH 9.6) overnight at 4 °C and blocked with 3% BSA in PBS with 0.05% Tween (PBST) for 1 h at room temperature (RT). Wells were washed 4 times with PBST and incubated with samples diluted in 0.5% BSA in PBST for 2 h at RT, followed by incubation with 0.25 μg/ml of the human TREM2 biotinylated detection antibody (R&D Systems, BAF1828) for 1 h at RT. After washing, samples were incubated with HRP-conjugated streptavidin (PerkinElmer, NEL750001EA, 1:10.000). The samples were washed and incubated with the chromogenic substrate TMB (3,3′,5,5′-tetramethylbenzidine) using Pierce TMB Substrate kit (Thermo Fisher Scientific, 34021). Upon optimal color development, reactions were stopped using 1 N HCL, and the optical density (O.D.) of the wells was read at 450 nM using the Epoch2 microplate reader (BioTek). The first batch of soluble TREM2 protein was quantified by Coomassie Brilliant Blue staining after electrophoresis in a 4–12% Bis-Tris gel with a standard curve of bovine serum albumin. These proteins were then used as standards for the quantification of the next batch of soluble TREM2 protein by ELISA. Subsequently, all quantifications were done by ELISA, and within each batch, a fraction of protein was stored to be used as ELISA standards for the following batches. We controlled for the presence of endotoxins using samples from different batches. To specifically detect TREM2^222^ TREM2^219^ by ELISA, we used the same protocol described above using different capture antibodies to coat the wells, Ab222 at 4 μg /ml and Ab219 at 2 μg/ml. The generation of ELISA standard curves and their interpolation was done using the BioTek Gen5 Analysis software.

### Electrophysiology

For these experiments, 4-month-old male mice were anesthetized and transcardially perfused with cold artificial cerebral spinal fluid (aCSF) (124 mM NaCl, 4.5 mM KCl, 1 mM MgCl_2_, 26 mM NaHCO_3_, 1.2 mM NaH_2_PO_4_, 10 mM glucose, 2 mM CaCl_2_) bubbled with 95% O_2_ and 5% CO_2_. The coronal slices (280 μm) were cut in an ice-cold sucrose-based solution using a vibratome Leica VT1200S. The slices were stored for 60 min in oxygenated aCSF at 30 °C and then kept at room temperature. Prior to recording slices were incubated for 60 min at room temperature in either a control-protein or active-protein solutions. The incubation system we used is like the one described by Hupp et al. [45]. We used 12 well-standard culture plates with a strainer basket inside. 95% O_2_ and 5% CO_2_ were bubbled through a fine tube placed between the basket and the wall of the plate without mechanically disturbing the slices. The volume of each well was 3 ml of aCSF in which the protein was dissolved to a concentration of 15 ng/ml. Heat-inactivated proteins (100 °C for 1 h) were used as controls.

The recordings were performed at 30–32 °C in a chamber that was perfused with oxygenated aCSF at a rate of about 2 mL/min. In some experiments, picrotoxin (50 μM) was added to the medium to block GABAA receptors. Field excitatory postsynaptic potentials (fEPSPs) were recorded using a Multiclamp 700B amplifier and Clampex software (Molecular Devices). Signals were low-pass filtered at 2 kHz and digitized at 50 kHz. Tungsten stereotrodes (~ 1 MΩ) were used to stimulate the Schaffer collaterals in the hippocampus CA1 region. Stimulation parameters were adjusted using a constant current isolated stimulator (Digitimer). An input-output curve was obtained, and then using the stimulation strength that produced about 50% of the maximum intensity, a stable baseline was recorded for 10 min stimulating at 0.05 Hz. Long-term potentiation (LTP) was induced by applying 4 100-Hz trains of 100 ms duration every 20 s. Changes in the slope of the response (mV/ms) fEPSPs were recorded for 60 min post-induction monitoring at 0.05 Hz. Data were expressed as a percentage of change with respect to the average baseline.

### Statistical analysis

Statistical analysis was performed using GraphPad Prism version 8 for Windows (GraphPad Software www.graphpad.com). Data were first analyzed for normality followed by statistical tests. The tests used were Student’s *t*-test or Mann-Whitney test.

## Results

### Expression of TREM2 transcripts increases in Alzheimer’s disease brain

Previous work studying the expression of specific *TREM2* transcripts in the brain of AD patients has not yielded consistent results. While some studies observed an increase of *TREM2* transcripts in the brain tissue of AD patients [17, 29], another study reports that *TREM2* transcript levels remain unaltered [16]. Importantly, these expression analyses were limited in terms of *TREM2* transcripts that were analyzed and/or the number of brain regions analyzed. Thus, we decided to apply different methods and models to understand how the expression of *TREM2* splice isoforms is affected in AD. We first analyzed the AMP-AD transcriptomic dataset and observed significant changes in particular brain regions (*P* < 0.05 and FDR < 0.1). The expression of *TREM2*^230^ in the superior frontal, parahippocampal, and inferior frontal gyrus of AD patients is increased (Fig. 1A). Furthermore, AD patients exhibit increased expression of *TREM2*^222^ in the temporal cortex and *TREM2*^219^ in the superior frontal and parahippocampal gyrus (Fig. 1A). We have also determined the relative amount of *TREM2* alternative isoforms in relation to the mean of the canonical transcript (Additional file 1: Fig. S1A). The median of both *TREM2*^222^ and *TREM2*^219^ relative expression is below 20% across brain regions with *TREM2*^222^ exhibiting the lowest levels. Furthermore, we designed specific primers for each *TREM2* isoform (Fig. 1B and Additional file 1: Fig. S1B) and observed a significant increase of these *TREM2* transcripts in post-mortem brain tissue (middle frontal gyrus) of a small cohort of control and AD patients by qPCR (Fig. 1C). In agreement, qPCR analysis of cortical brain tissue of 7-month-old 5xFAD mice expressing the human *TREM2* gene revealed that all three transcripts were increased in the AD mouse brain (Fig. 1D). Although endogenous *Trem2* splicing in mice is distinct from humans, similar results were found for the murine endogenous *TREM2* isoforms in the cortex of two amyloid models, 5xFAD (6-month-old animals) and APPPS1 (4-month-old animals), namely *Trem2*^227^ (ENSMUST00000024791) which encodes the full-length receptor and *Trem2*^249^ (ENSMUST00000113237) which lacks the transmembrane domain [46] (Additional file 1: Fig. S1C-D).

### Generation of soluble TREM2 from TREM2 isoforms

**Fig. 2:**
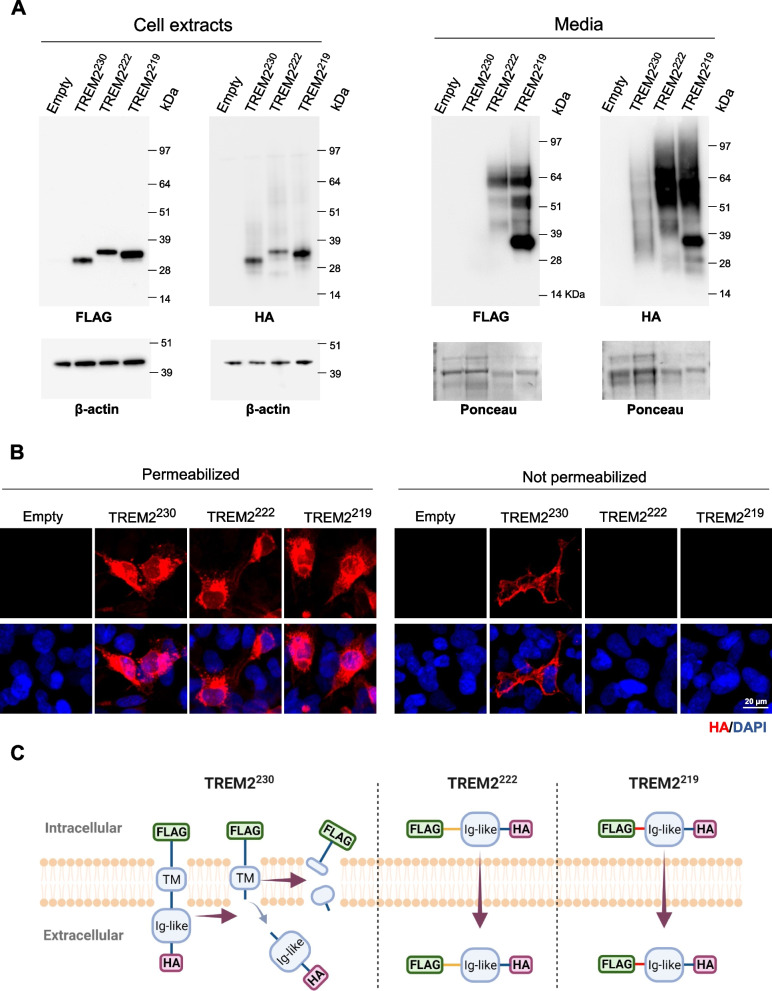
Secretion of TREM2 isoforms. **A** Western blot analysis of TREM2 isoforms C-terminus (FLAG-tagged) and N-terminus (HA-tagged) in cell extracts (left panel) and media (right panel) of HEK-293T transfected with tagged TREM2 isoforms. The FLAG and HA antibodies were used to detect the C- and N-terminus, respectively. β-Actin and Ponceau staining were used as loading controls for cell extracts and media, respectively. **B** Immunocytochemistry in permeable and non-permeable conditions (presence and absence of detergents, respectively) was performed in HMC3 cells transfected with TREM2-tagged isoforms. The HA antibody was used to detect all TREM2 isoforms and DAPI to stain cell nuclei. **C** Scheme of the processing of TREM2 isoforms based on the transfection model

To analyze the processing and secretion of TREM2 isoforms, we designed constructs encoding for TREM2^230^, TREM2^222^, or TREM2^219^ fused to a C-terminal FLAG tag and N-terminal HA tag with a linker sequence. Human HEK-293T cells were transfected with these plasmids, and the expression of each isoform was analyzed in the cell extracts and media by western blot. Both FLAG and HA tags were detected within cell extracts for all isoforms (Fig. 2A). TREM2^230^ receptor is cleaved at the stalk region (H157) by ADAM proteinases, releasing the extracellular N-terminal fragment (sTREM2^H157^) and retaining the intracellular C-terminal. Thus, as expected, we only detected the N-terminal HA tag and not the C-terminal FLAG tag in the media from cells transfected with TREM2^230^ (Fig. 2A). However, both C-terminal FLAG and N-terminal HA tags were detected for TREM2^222^ and TREM2^219^ at similar molecular weights as the ones observed in the cell extracts (Fig. 2A), supporting that these two isoforms are secreted full-length to the extracellular milieu (sTREM2^222^ and sTREM2^219^). It is noteworthy that a high-molecular-weight smear is detected for all three sTREM2 species, suggesting robust glycosylation of sTREM2, which has been previously described [12, 47]. Immunocytochemistry was also performed in transfected human microglial cell line HMC3 with permeabilizing and non-permeabilizing conditions. The three TREM2 isoforms can be visualized within the cells that have been permeabilized; however, in non-permeabilized conditions, we could only detect fluorescent signal in TREM2^230^-transfected cells (Fig. 2B). These data suggest that TREM2^230^ resides intracellularly and at the cellular membrane level, and is cleaved to generate a soluble N-terminal fragment (Fig. 2C). On the other hand, TREM2^222^ and TREM2^219^ localize preferentially intracellularly and are secreted full-length to the extracellular milieu. These findings are consistent with the disruption of the transmembrane domain observed in the TREM2 splice isoforms. Although endogenous *Trem2* splicing in mice is distinct from humans, similar results were found for the murine TREM2 isoforms (Additional file 1: Fig. S2A-2B).

### Soluble TREM2 species inhibit LTP

**Fig. 3:**
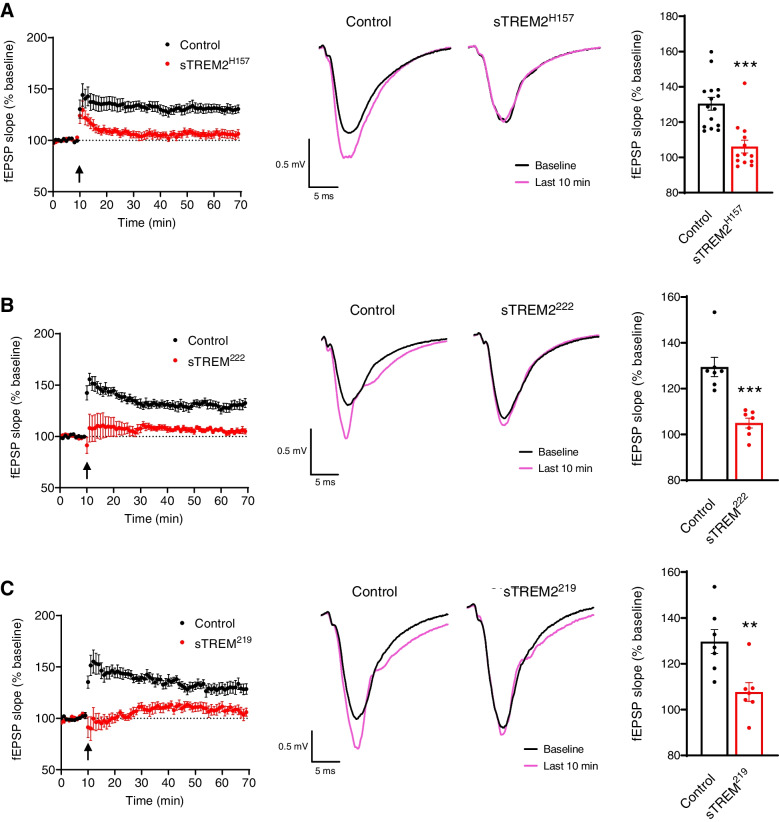
Soluble TREM2 species inhibit long-term potentiation (LTP). Electrophysiology analysis of LTP in C57BL/6J mice brain slices incubated with soluble TREM2 species at 15 ng/ml. **A–C** Incubations with sTREM2^H157^, sTREM2^222^, and sTREM2^219^, respectively. For each isoform, a time course with the average fESP slopes (% baseline) is shown with an arrow that indicates the time of stimulation (left panel) accompanied by a representative fEPSP trace (middle panel) and the quantification of the last 10 min of fESP slope measurements (% baseline) (right panel). **A** Fifteen recordings from 10 animals for control and 13 recordings from 6 animals for sTREM2^H157^. **B**, **C** Seven recordings from 6 animals for both control and proteins. Statistical analysis was performed by the Mann-Whitney tests for sTREM2^H157^ and sTREM2^222^ and the Student *t*-test for sTREM2^219^. Data are expressed as mean values ± SEM (**P < 0.01; ***P < 0.001)

**Fig. 4:**
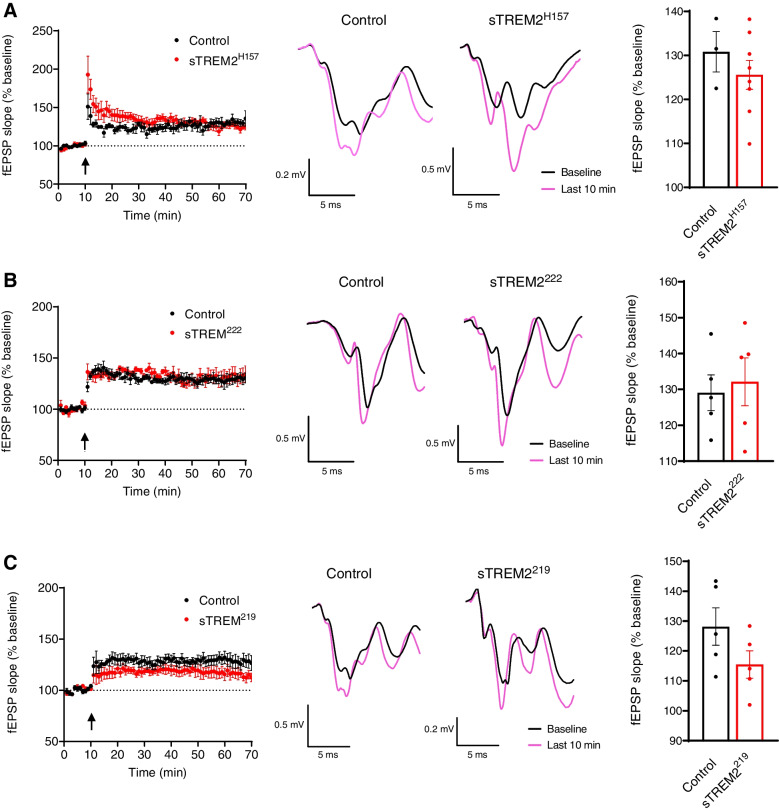
Inhibition of GABAA receptors abolishes the effect of soluble TREM2 species on long-term potentiation (LTP). Electrophysiology analysis of LTP in C57BL/6J mice brain slices incubated with soluble TREM2 species at 15 ng/ml and picrotoxin 50 μM. **A–C** Incubations with sTREM2^H157^, sTREM2^222^, and sTREM2^219^, respectively. For each isoform, a time course with the average fESP slopes (% baseline) is shown with an arrow that indicates the time of stimulation (left panel) accompanied by a representative fEPSP trace (middle panel) and the quantification of the last 10 min of fESP slope measurements (% baseline) (right panel). **A** Three slices from 2 animals for control and 8 slices from 6 animals for sTREM2^H157^ were recorded. **B**, **C** Five slices from 5 animals for both control and proteins were recorded. Statistical analysis was performed by the Student *t*-tests. Data are expressed as mean values ± SEM

Previous reports support an interaction between sTREM2 and neurons [27, 28]; however, its endogenous effects on neuronal function remain unclear. Thus, we sought to analyze the effect of sTREM2 species on synaptic plasticity in mouse brain slices using a concentration within the range reported for human CSF [19–25, 32]. To produce sTREM2 species from each isoform, we transfected the human cell line HEK-293T with the previously described constructs encoding TREM2. We purified these soluble species from cell media using the N-terminal HA tag and visualized them by silver staining (Additional file 1: Fig. S3A). The pattern observed for each sTREM2 species with the silver staining is identical to the one detected by western blot of cell media (Fig. 1A), supporting that the purification method has not abruptly changed the structure or post-translational modifications of these sTREM2 species. To study the endogenous actions of sTREM2 on LTP, we incubated brain slices of 3–4-month-old C57BL/6J mice with 15 ng/ml of each sTREM2 isoform, which is a concentration within the range reported for human CSF. Heat-inactivated proteins were used as a control. Each of the three sTREM2 species dramatically inhibited LTP (Fig. 3). To confirm that there were no soluble factors derived from HEK cells interfering with the LTP, the media from HEK cells transfected with an empty plasmid was subjected to the purification protocol and had no effect on LTP compared to untreated slices (Additional file 1: Fig. S3B), supporting that LTP is inhibited specifically by the presence of sTREM2 species. This inhibitory effect of sTREM2 species was conserved when LTP was measured in slices of the amyloidogenic 5xFAD model (Additional file 1: Fig. S3C-E), suggesting sTREM2 actions on LTP are independent of the presence of plaque pathology. We have also purified the murine sTREM2 species and observed they both inhibit LTP induction as well (Additional file 1: Fig. S4A-B), suggesting that the biological activity of sTREM2 is similar between humans and mice. Interestingly, sTREM2 species were unable to exert this inhibitory effect on LTP when concomitantly incubated with the GABAA receptor antagonist picrotoxin at 50 μM (Fig. 4).

### Immunodetection of TREM2 splice isoforms in human brain extracts

**Fig. 5:**
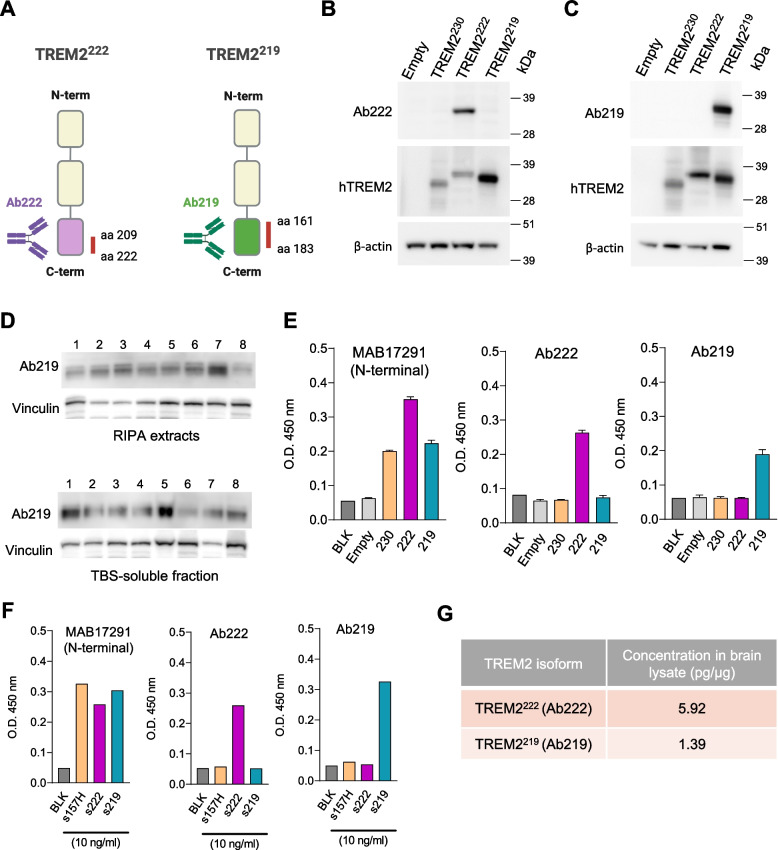
Detection of TREM2 alternative isoforms protein expression in the human brain. **A** Design of customized antibodies to specifically detect human TREM2^222^ (Ab222) and TREM2^219^ (Ab219). **B**, **C** Western blot of TREM2^222^ and TREM2^219^ in cell extracts from HEK-293T transfected with tagged TREM2 isoforms (TREM2^230^, TREM2^222^, and TREM2^219^) using Ab222 (**B**), Ab219 (**C**), anti-human TREM2, and anti-β-actin antibodies. **D** Western blot analysis of TREM2^219^ expression in RIPA (upper blot) and TBS-soluble extracts (bottom blot) from the brain tissue of control human subjects (CTRL) and patients with Alzheimer’s disease (AD) using Ab219 and vinculin as a loading control. **E** ELISA of lysates from HMC3 cells transfected with TREM2 isoforms or empty plasmid (*n* = 3). The capture antibodies used were MAB17291 (N-terminal), Ab222, and Ab219. Data are shown as mean values ± SEM of optical density at 450 nm (O.D. 450 nm). **F** ELISA of purified soluble TREM2 proteins (10 ng/ml) using a TREM2 N-terminal capture antibody (MAB17291), as well as Ab222 and Ab219 to assess their specificity. Data are shown as O.D. 450 nm. **G** Concentration of TREM2^222^ and TREM2^219^ in a pool of human brain lysate determined by ELISA

To understand if the alternatively spliced transcripts (TREM2^222^ and TREM2^219^) are translated into protein in the human brain, we developed customized antibodies that specifically detect each of these isoforms (Fig. 5A—Ab222 and Ab219) and validated their specificity by analyzing transfected HEK cells with the different TREM2 isoforms (Fig. 5B). We were able to specifically detect TREM2^219^ in TBS-soluble and RIPA brain extracts from humans, although the molecular weight is slightly lower compared to the tagged constructs transfected into HEK cells (Additional file 1: Fig. S4A and B). Although Ab222 showed specificity for TREM2^222^ in transfected cells, it failed to recognize a specific band on the western blot of human samples (Additional file 1: Fig. S5C). Nonetheless, we developed sandwich ELISA assays using Ab222 and Ab219 as capture antibodies and the N-terminal TREM2 antibody (BAF1828) for the detection of these isoforms. To analyze the specificity of these antibodies for ELISA, we used lysates from HMC3 cells transfected with TREM2 isoforms or empty plasmid. To validate the transfection, we used MAB17291 (R&D) as the capture antibody which is a TREM2 N-terminal antibody and detects all transfected isoforms. As expected, using MAB17291, we detected a positive signal (OD 450 nm above Blank and Empty) in the extracts from cells transfected with any of the TREM2 isoforms (Fig. 5E). Importantly, our results show that the ELISA assays using Ab222 and Ab219 as capture antibodies only detected a positive signal in the cell extracts transfected with TREM2^222^ and TREM2^219^, respectively (Fig. 5E). Additionally, we also tested the different purified soluble TREM2 species at the same concentration (10 ng/ml) and confirmed the specificity of Ab222 and Ab219 for soluble TREM2^222^ and TREM2^219^ (Fig. 5F). These ELISA assays were used to detect the presence of both TREM2^222^ and TREM2^219^ in a sample of pooled human brain RIPA lysates (Fig. 5G). The sample dilutions were selected based on dilution linearity and spike recovery percentage (Additional file 1: Fig. S5D). For the detection of TREM2^222^ and TREM2^219^, the standard curves were prepared with a 2-fold serial dilution and ranged from 2500 to 312.5 pg/ml and 1250 to 78.125 pg/ml, respectively (Additional file 1: Fig. S5E). Altogether, we provide evidence that TREM2^222^ and TREM2^219^ isoforms are both translated in the human brain.

## Discussion

TREM2 encodes a transmembrane receptor that, in the brain, is specifically expressed by microglia and modulates their function [9]. Genetic variants have been associated with an elevated risk of AD and other neurodegenerative diseases, and studies in animal models have uncovered the molecular mechanisms subserving the actions of this receptor [9, 10, 48]. TREM2 is also present as a soluble protein (sTREM2), and most studies have focused on sTREM2 as a potential biomarker for AD progression [19–26], while very few studies have explored sTREM2 biological functions [30, 31, 49, 50]. Specifically, sTREM2 has been reported to activate the microglia and ameliorate the pathology in 5xFAD mice when administered at significantly higher, non-physiological, levels compared to human CSF [30, 31]. Although the concentration of sTREM2 used in these studies may be relevant for therapeutic strategies that focus on delivering the protein, the endogenous actions of sTREM2 on neuronal function remain unclear. In addition, the source of sTREM2 in the human brain and CSF also remains elusive. The production of sTREM2 is often attributed to the cleavage of TREM2^230^, releasing the fragment sTREM2^H157^; however, it has been suggested that it can also derive from *TREM2* splice isoforms *TREM2*^*222*^ and *TREM2*^*219*^ [13–15]. Our results demonstrate that the expression of these alternative splice transcripts, as well as the canonical *TREM2*^*230*^, increase in the brains of patients with AD. Experimental validation that *TREM2* splice isoforms produce sTREM2 has been lacking; however, the present work provides evidence that splice isoforms *TREM2*^*222*^ and *TREM2*^*219*^ contribute to the sTREM2 pool. Furthermore, our analysis revealed that each of the soluble species of TREM2 exerts an inhibitory effect on synaptic plasticity. Importantly, we were able to detect TREM2^222^ and TREM2^219^ protein expression in post-mortem human brain tissue using immunoassays, supporting that *TREM2* splice transcripts are translated into functional proteins in the human brain.

Our findings showing increased expression of *TREM2* transcripts in the AD brain agree with previous studies. It has been reported that overall *TREM2* transcripts (no distinction of transcripts) and specifically *TREM2*^*219*^ are increased in the temporal cortex of patients with AD [17]; however, these authors did not differentially analyze *TREM2*^230^ and *TREM2*^*222*^ transcripts. Another study revealed increased expression of *TREM2*^*230*^ in AD brain tissue by qPCR and RNAscope analysis [29]. In contrast to our results, a recent RNA-Seq meta-analysis of three AD datasets reported that overall *TREM2*, *TREM2*^230^, and *TREM2*^*222*^ transcripts were not increased in AD, although there was a nominal association between increased expression of *TREM2*^*219*^ and AD cases [16]. Furthermore, these authors found that *TREM2*^*222*^ and *TREM2*^*219*^ are expressed at approximately 60% of the levels of the canonical isoform *TREM2*^*230*^. However, we observed that median expression levels of *TREM2*^*222*^ and *TREM2*^*219*^ relative to *TREM2*^230^ are below 20%. These discrepancies are likely related to the use of different datasets and the fact that the authors restricted their expression analysis solely to the BM36 parahippocampal region. Interestingly, in the case of *TREM2*^*219*^ relative expression, our data agree with a different study which reports that *TREM2*^*219*^ is expressed 5–7 times less than the overall *TREM2* levels [17]. Importantly, the relative abundance of *TREM2* transcripts may not directly correlate to the relative abundance of sTREM2 species. For instance, the TREM2^230^ receptor needs to be cleaved to produce sTREM2 unlike the alternative splice isoforms; thus, although the *TREM2*^230^ transcript is more highly expressed than the alternative isoforms, it does not necessarily mean it contributes more to the sTREM2 pool.

Our results demonstrate that TREM2 splice isoforms TREM2^222^ and TREM2^219^ are secreted full-length (sTREM2^222^ and sTREM2^219^), while TREM2^230^ releases its cleaved N-terminus fragment sTREM2^H157^. The full sequence of the sTREM2^H157^ is identical to the N-terminal sequence of sTREM2^222^ and sTREM2^219^; however, unlike sTREM2^H157^, the sTREM2^222^ and sTREM2^219^ proteins retain their distinct C-termini upon secretion. Importantly, these data support that the sTREM2 pool in the human brain is heterogeneous, composed of different sTREM2 proteins. Furthermore, our results suggest that these sTREM2 proteins are subjected to substantial post-translational modifications (PTMs) upon secretion, most prominently glycosylation, in agreement with previous reports [12, 47]. The identification of these PTMs and the analysis of how they affect the biological actions of sTREM2 species will be an important extension of this study, as well as the characterization of the pathways that regulate the secretion of TREM2^222^ and TREM2^219^.

The present work demonstrates that concentrations of sTREM2^H157^, sTREM2^222^, and sTREM2^219^ in the range of what has been reported in the CSF of AD patients act to inhibit LTP independently of the presence of amyloid plaques and related pathology. It is interesting that blocking GABAA receptors prevented the LTP-impairing actions of sTREM2. This could be interpreted as sTREM2’s actions requiring the activation of GABAA receptors. Alternatively, the removal of tonic GABA transmission could overcome some inhibitory impact of sTREM2 on neuronal excitability, thus enhancing LTP induction. Nonetheless, whether sTREM2 acts directly on neurons (interacting with GABAA receptors, other synaptic proteins, or modulators of neuronal excitability) or indirectly through other cell types will be assessed in future studies. sTREM2 proteins have been shown to bind to neurons, which argues in favor of a direct neuronal mechanism [27, 28], but it has also been reported it can act through the microglia to increase LTP in 5xFAD mice [30, 31]. However, this latter finding was observed using sTREM2 protein at 50 nM (≈ 1000 ng/ml) which is dramatically higher than what has been reported in the CSF of patients with AD (≈ 0.1–25 ng/ml) [19–25, 32]. The high concentration of sTREM2 used by these authors could be the reason for the discrepancy with our findings showing an inhibition of LTP with all sTREM2 species, as we used significantly lower concentrations to better reflect human CSF levels (15 ng/ml). The use of different cell systems to produce the proteins and purification processes may have also contributed to this disparity. Our results suggest there is an increased production of sTREM2 species in the AD brain, which might act to disrupt neuronal plasticity. Nonetheless, in vivo studies specifically focused on soluble TREM2 will be necessary to further study the exact role of sTREM2 species on synaptic function and AD progression.

We provide evidence suggesting that TREM2 alternatively spliced isoforms are translated and expressed in the human brain. We obtained customized antibodies to specifically detect TREM2^222^ and TREM2^219^ and used them in western blot analysis of post-mortem brain extracts of control and AD cases. The customized antibody for TREM2^222^ (Ab222) was shown to be specific with transfected HEK cell extracts; however, we could not confidently recognize a specific band for this protein in human brain extracts by western blot. However, we were able to detect the protein expression of *TREM2*^*222*^ in the brain tissue using an ELISA assay. TREM2^219^ was detected by ELISA and western blot. Although we managed to detect TREM2 isoforms using these methods, further characterization and optimization of these immunoassays will be performed to further validate our findings. The present results suggest that transcripts derived from alternative splicing of *TREM2* are indeed translated in the human brain and can contribute to a heterogeneous pool of sTREM2 with relevant biological actions. In agreement with our results, TREM2^219^ (Q9NZC2-2) has been detected by mass spectrometry analysis in the CSF of human AD patients and reported in a recent preprint [51].

## Conclusions

The expression of the *TREM2* transcripts *TREM2*^230^, *TREM2*^222^, and *TREM2*^219^ is increased in the brain of AD patients. Our findings suggest that all three isoforms can produce different soluble TREM2 that globally act to inhibit LTP through a mechanism that requires the activation of GABAA receptors. Thus, the production of sTREM2 species may be linked to a disruption of synaptic plasticity in AD and possibly other diseases. These findings provide novel insight into the generation, regulation, and function of soluble TREM2, which fits into the complex biology of TREM2 and its role in human health and disease.

## Supplementary Information

**Additional file 1: Table S1.** Information of post-mortem samples obtained from Johns Hopkins. **Table S2.** List of primers **Table S3.** Nucleotide sequence TREM2 HA- and FLAG-tagged constructs **Figure S1.** Relative expression of *TREM2* alternative isoforms, specificity of *TREM2* primers and expression of *Trem2* splice isoforms amyloidogenic mouse models. **Figure S2.** Secretion of murine TREM2 isoforms. **Figure S3.** Soluble TREM2 species inhibit long-term potentiation (LTP) in brain slices of amyloidogenic 5xFAD mice. **Figure S4.** Murine soluble TREM2 species inhibit LTP. **Figure S5.** Western blot of TREM2^222^ and TREM2^219^, ELISA dilutions and standard curves. **Figure S6.** Uncropped western blots.

## Data Availability

RNA-Seq data were downloaded from the Accelerating Medicines Partnership for Alzheimer’s Disease (AMP-AD) Consortium (https://adknowledgeportal.synapse.org/Explore/Programs/DetailsPage?Program=AMP-AD). The raw data from ROS/MAP cohort (https://www.synapse.org/#!Synapse:syn8456638) [17], Mayo cohort (https://www.synapse.org/#!Synapse:syn8466816) [18], and MSBB cohort (https://www.synapse.org/#!Synapse:syn8485017) [19] are accessible through the Synapse database (https://www.synapse.org/).
