# Supplementary material for: TREM2 splice isoforms generate soluble TREM2 species that disrupt long-term potentiation
Source: Genome Med. 2023 Feb 20;15:11. doi: 10.1186/s13073-023-01160-z (PMC9940368; doi:10.1186/s13073-023-01160-z)
Supplement: Supplementary file 1 — Additional file 1: Table S1. Information of post-mortem samples obtained from Johns Hopkins. Table S2. List of primers Table S3. Nucleotide sequence TREM2 HA- and FLAG-tagged constructs Figure S1. Relative expression of TREM2 alternative isoforms, specificity of TREM2 primers and expression of Trem2 splice isoforms amyloidogenic mouse models. Figure S2. Secretion of murine TREM2 isoforms. Figure S3. Soluble TREM2 species inhibit long-term potentiation (LTP) in brain slices of amyloidogenic 5xFAD mice. Figure S4. Murine soluble TREM2 species inhibit LTP. Figure S5. Western blot of TREM2222 and TREM2219, ELISA dilutions and standard curves. Figure S6. Uncropped western blots. [file 13073_2023_1160_MOESM1_ESM.docx]

**Table S1. Information of post-mortem samples obtained from Johns Hopkins**

| **Diagnosis (n)** | **CERAD (n)** | **Braak (n)** | **Age**  **(average ± stdev)** | **Sex (n)** | **Race (n)** |
| --- | --- | --- | --- | --- | --- |
| Control (10) | - | - | 71.6 ± 8.6 | F (5)  M (5) | W (9)  AA (1) |
| AD (12) | C (10)  B (2) | 6 (10)  5 (1)  4 (1) | 76.3 ± 13.1 | F (8)  M (4) | W (12) |

**Table S2. List of primers**

| Gene | Forward (5’-3’) | Reverse (5’-3’) |
| --- | --- | --- |
| *TREM2*^230^ | GCATCTCCAGGAGCCTCTTG | TGAGAAAGATGCAGGCCAGG |
| *TREM2*^222^ | CATCTCCAGGCCATCTCAAGGCTCCCATCTGC | CAGGAGGAGAAGGATGGAAGTG |
| *TREM2*^219^ | CGGGATGCTGGAGATCTCTG | TCTCAGCCCTGGAGATGCT |
| *GAPDH* | ACAACTTTGGTATCGTGGAAGG | GCCATCACGCCACAGTTTC |
| *Trem2*^227^ | CAGCACCTCCAGGAATCAAGA | GAGAAGAATGGAGGTGGGTGG |
| *Trem2*^249^ | AACACAGCACCTCCAGGCA | TGGTAGGCTAGAGGTGACCCA |
| *Gapdh* | CCCTCACAATTTCCATCCG | TCCCTAGGCCCCTCCTGTTA |

**Table S3. Nucleotide sequence** **TREM2 HA- and FLAG-tagged constructs**

| Plasmid | Peptide nucleotide sequence |
| --- | --- |
| *TREM2*^230^ | ATGGAGCCTCTCCGGCTGCTCATCTTACTCTTTGTCACAGAGCTGTCCGGAGCCCATTACCCATACGATGTTCCAGATTACGCTTCTGGTGGCGGTGGCGGACTTGAGCACAACACCACAGTGTTCCAGGGCGTGGCGGGCCAGTCCCTGCAGGTGTCTTGCCCCTATGACTCCATGAAGCACTGGGGGAGGCGCAAGGCCTGGTGCCGCCAGCTGGGAGAGAAGGGCCCATGCCAGCGTGTGGTCAGCACGCACAACTTGTGGCTGCTGTCCTTCCTGAGGAGGTGGAATGGGAGCACAGCCATCACAGACGATACCCTGGGTGGCACTCTCACCATTACGCTGCGGAATCTACAACCCCATGATGCGGGTCTCTACCAGTGCCAGAGCCTCCATGGCAGTGAGGCTGACACCCTCAGGAAGGTCCTGGTGGAGGTGCTGGCAGACCCCCTGGATCACCGGGATGCTGGAGATCTCTGGTTCCCCGGGGAGTCTGAGAGCTTCGAGGATGCCCATGTGGAGCACAGCATCTCCAGGAGCCTCTTGGAAGGAGAAATCCCCTTCCCACCCACTTCCATCCTTCTCCTCCTGGCCTGCATCTTTCTCATCAAGATTCTAGCAGCCAGCGCCCTCTGGGCTGCAGCCTGGCATGGACAGAAGCCAGGGACACATCCACCCAGTGAACTGGACTGTGGCCATGACCCAGGGTATCAGCTCCAAACTCTGCCAGGGCTGAGAGACACGGGTGGTTCTGGAGACTACAAAGACGATGACGACAAGTGA |
| *TREM2*^222^ | ATGGAGCCTCTCCGGCTGCTCATCTTACTCTTTGTCACAGAGCTGTCCGGAGCCCATTACCCATACGATGTTCCAGATTACGCTTCTGGTGGCGGTGGCGGACTTGAGCACAACACCACAGTGTTCCAGGGCGTGGCGGGCCAGTCCCTGCAGGTGTCTTGCCCCTATGACTCCATGAAGCACTGGGGGAGGCGCAAGGCCTGGTGCCGCCAGCTGGGAGAGAAGGGCCCATGCCAGCGTGTGGTCAGCACGCACAACTTGTGGCTGCTGTCCTTCCTGAGGAGGTGGAATGGGAGCACAGCCATCACAGACGATACCCTGGGTGGCACTCTCACCATTACGCTGCGGAATCTACAACCCCATGATGCGGGTCTCTACCAGTGCCAGAGCCTCCATGGCAGTGAGGCTGACACCCTCAGGAAGGTCCTGGTGGAGGTGCTGGCAGACCCCCTGGATCACCGGGATGCTGGAGATCTCTGGTTCCCCGGGGAGTCTGAGAGCTTCGAGGATGCCCATGTGGAGCACAGCATCTCCAGGCCATCTCAAGGCTCCCATCTGCCTTCTTGTCTCTCCAAGGAGCCTCTTGGAAGGAGAAATCCCCTTCCCACCCACTTCCATCCTTCTCCTCCTGGCCTGCATCTTTCTCATCAAGATTCTAGCAGCCAGCGCCCTCTGGGCTGCAGCCTGGCATGGACAGAAGCCAGGGACACATCCACCCAGGGTGGTTCTGGAGACTACAAAGACGATGACGACAAGTGA |
| *TREM2*^219^ | ATGGAGCCTCTCCGGCTGCTCATCTTACTCTTTGTCACAGAGCTGTCCGGAGCCCATTACCCATACGATGTTCCAGATTACGCTTCTGGTGGCGGTGGCGGACTTGAGCACAACACCACAGTGTTCCAGGGCGTGGCGGGCCAGTCCCTGCAGGTGTCTTGCCCCTATGACTCCATGAAGCACTGGGGGAGGCGCAAGGCCTGGTGCCGCCAGCTGGGAGAGAAGGGCCCATGCCAGCGTGTGGTCAGCACGCACAACTTGTGGCTGCTGTCCTTCCTGAGGAGGTGGAATGGGAGCACAGCCATCACAGACGATACCCTGGGTGGCACTCTCACCATTACGCTGCGGAATCTACAACCCCATGATGCGGGTCTCTACCAGTGCCAGAGCCTCCATGGCAGTGAGGCTGACACCCTCAGGAAGGTCCTGGTGGAGGTGCTGGCAGACCCCCTGGATCACCGGGATGCTGGAGATCTCTGGTTCCCCGGGGAGTCTGAGAGCTTCGAGGATGCCCATGTGGAGCACAGCATCTCCAGGGCTGAGAGACACGTGAAGGAAGATGATGGGAGGAAAAGCCCAGGAGAAGTCCCACCAGGGACCAGCCCAGCCTGCATACTTGCCACTTGGCCACCAGGACTCCTTGTTCTGCTCTGGCAAGAGACTACTCTGCCTGAACACTGCTTCTCCTGGACCCTGGAAGCAGGGACTGGTGGTGGTTCTGGAGACTACAAAGACGATGACGACAAGTGA |
| *Trem2*^227^ | ATGGGACCTCTCCACCAGTTTCTCCTGCTGCTGATCACAGCCCTGTCCCAAGCCCTTTACCCATACGATGTTCCAGATTACGCTTCTGGTGGCGGTGGCGGACTTGAGCTCAACACCACGGTGCTGCAGGGCATGGCCGGCCAGTCCTTGAGGGTGTCATGTACTTATGACGCCTTGAAGCACTGGGGGAGACGCAAGGCCTGGTGTCGGCAGCTGGGTGAGGAGGGCCCATGCCAGCGTGTGGTGAGCACACACGGTGTGTGGCTGCTGGCCTTCCTGAAGAAGCGGAATGGGAGCACAGTCATCGCAGATGACACCCTTGCTGGAACCGTCACCATCACTCTGAAGAACCTCCAAGCCGGTGACGCGGGCCTCTACCAGTGTCAGAGTCTCCGAGGCCGAGAGGCTGAGGTCCTGCAGAAAGTACTGGTGGAGGTGCTGGAGGACCCTCTAGATGACCAAGATGCTGGAGATCTCTGGGTCCCCGAGGAGTCATCGAGTTTCGAGGGTGCCCAAGTGGAACACAGCACCTCCAGGAATCAAGAGACCTCCTTCCCACCCACCTCCATTCTTCTCCTCCTGGCCTGCGTTCTCCTGAGCAAGTTTCTTGCAGCCAGCATCCTCTGGGCTGTGGCCAGGGGCAGGCAGAAGCCGGGAACACCTGTGGTCAGAGGGCTGGACTGTGGCCAAGATGCTGGGCACCAACTTCAGATCCTCACTGGACCCGGAGGTACGGGTGGTTCTGGAGACTACAAAGACGATGACGACAAGTGA |
| *Trem2*^249^ | ATGGGACCTCTCCACCAGTTTCTCCTGCTGCTGATCACAGCCCTGTCCCAAGCCCTTTACCCATACGATGTTCCAGATTACGCTTCTGGTGGCGGTGGCGGACTTGAGCTCAACACCACGGTGCTGCAGGGCATGGCCGGCCAGTCCTTGAGGGTGTCATGTACTTATGACGCCTTGAAGCACTGGGGGAGACGCAAGGCCTGGTGTCGGCAGCTGGGTGAGGAGGGCCCATGCCAGCGTGTGGTGAGCACACACGGTGTGTGGCTGCTGGCCTTCCTGAAGAAGCGGAATGGGAGCACAGTCATCGCAGATGACACCCTTGCTGGAACCGTCACCATCACTCTGAAGAACCTCCAAGCCGGTGACGCGGGCCTCTACCAGTGTCAGAGTCTCCGAGGCCGAGAGGCTGAGGTCCTGCAGAAAGTACTGGTGGAGGTGCTGGAGGACCCTCTAGATGACCAAGATGCTGGAGATCTCTGGGTCCCCGAGGAGTCATCGAGTTTCGAGGGTGCCCAAGTGGAACACAGCACCTCCAGGCAGGTTTCATCCTGTGGGTCACCTCTAGCCTACCACCTTCCTCCTCTTTCCAAGGAATCAAGAGACCTCCTTCCCACCCACCTCCATTCTTCTCCTCCTGGCCTGCGTTCTCCTGAGCAAGTTTCTTGCAGCCAGCATCCTCTGGGCTGTGGCCAGGGGCAGGCAGAAGCCGGGAACACCTGTGGTCAGAGGGCTGGACTGTGGCCAAGATGCTGGGCACCAACTTCAGATCCTCACTGGACCCGGAGGTACGTGAGAGAATTCGGTGGTTCTGGTGACTACAAAGACGATGACGACAAGTGA |

**
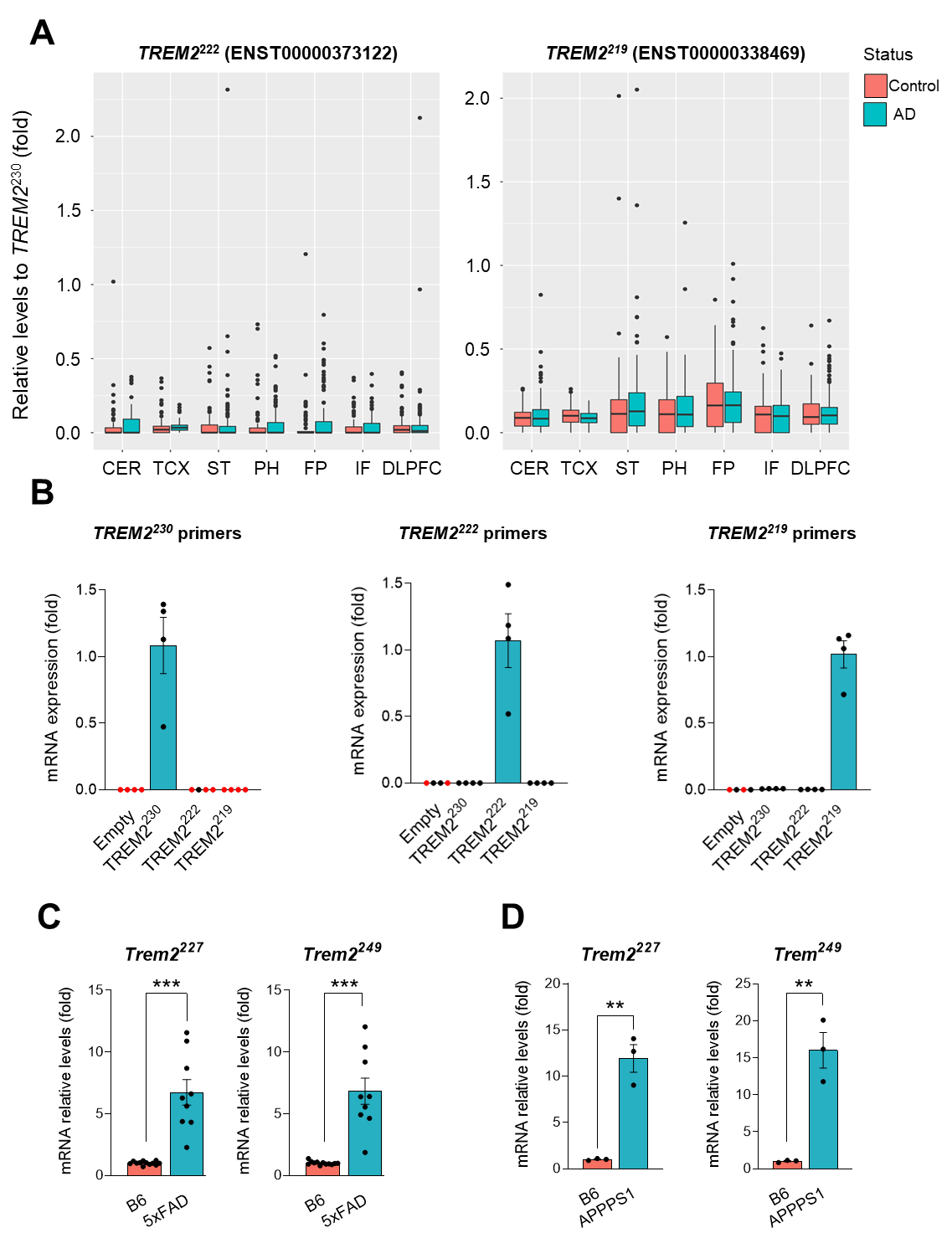
**

**Figure S1. Relative expression of *TREM2* alternative isoforms, specificity of *TREM2* primers and expression of *Trem2* splice isoforms amyloidogenic mouse models. A)** Expression of *TREM2*^222^ and *TREM2*^219^ relative to *TREM2*^230^ in different brain regions control subjects (Control) and patients with late-onset Alzheimer’s disease (AD) using AMP-AD dataset. **B)** qPCR analysis of human *TREM2* isoforms (*TREM2*^230^, *TREM2*^222^ and *TREM2*^219^) in transfected HEK-293T using primers specifically designed for each isoform. Non-detectable signal in the qPCR was plotted as a red circle with the value “0” on x-axis. **C)** and **D)** Expression of the murine *Trem2* splice isoforms (*Trem2*^227^ and *Trem2*^249^) in the cortical extracts of the 6-month-old 5xFAD **(C)** and 4-month-old APPPS1 **(D)** mice compared to non-transgenic controls (B6). Both sexes were analyzed for the 5xFAD mice (5 males and 7 females B6; 5 males and 4 females 5xFAD). Analysis of APPPS1 was done using females (n=3 per genotype). Statistical analysis was performed by Student t-test. Data are expressed as mean values ± SEM (**P*<0.05 ** *P*<0.01 *** *P*<0.001).

**
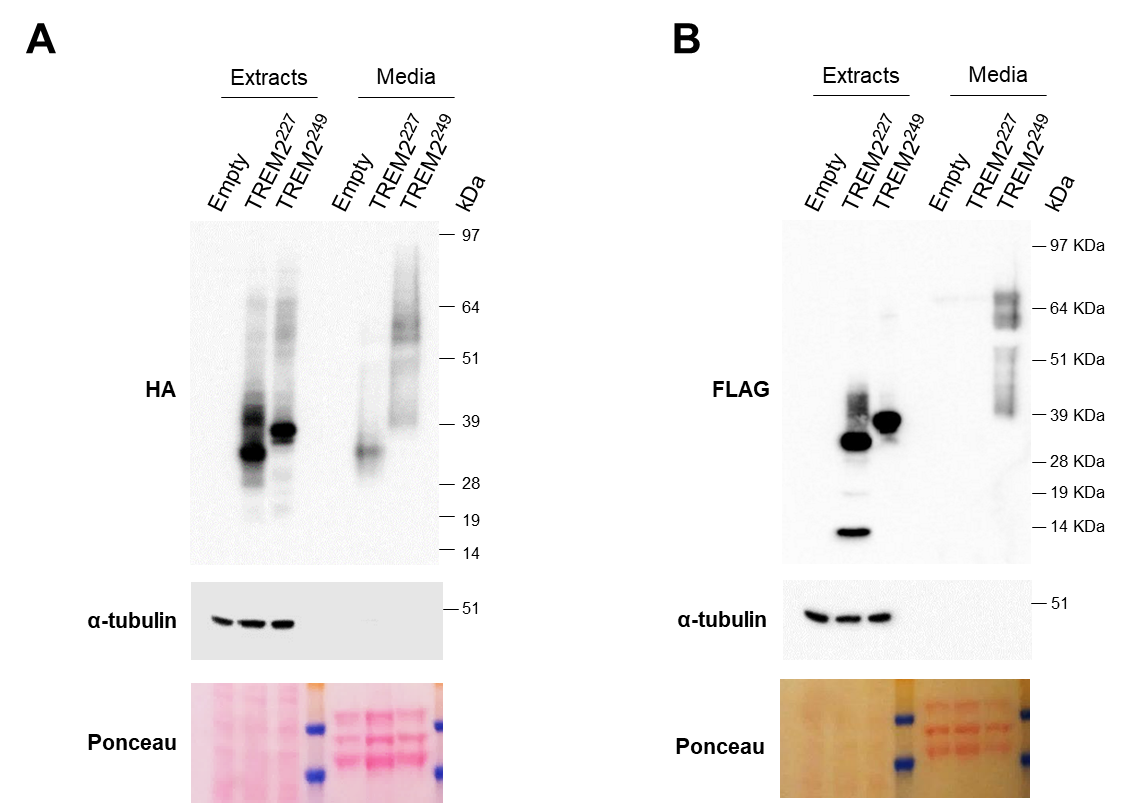
**

**Figure S2. Secretion of murine TREM2 isoforms.** Western blot analysis of TREM2 murine isoforms (TREM2^227^ and TREM2^249^) C-terminus (FLAG-tagged) and N-terminus (HA-tagged) in cell extracts (left panel) and media (right panel) of HEK-293T transfected with tagged TREM2 isoforms. The HA **(A)** and FLAG **(B)** antibodies were used to detect the C- and N-terminus, respectively.


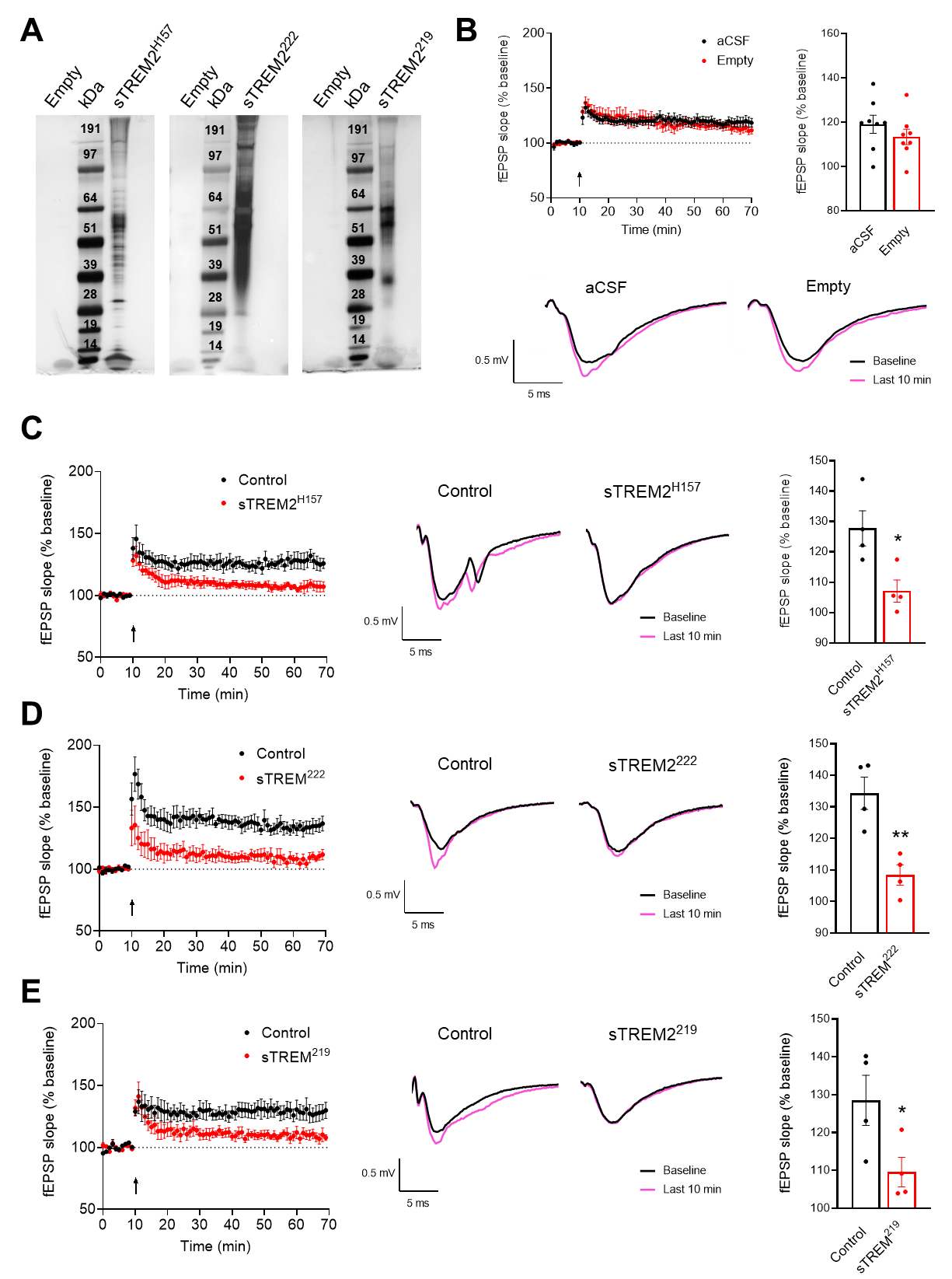


**Figure S3. Soluble TREM2 species inhibit long-term potentiation (LTP) in brain slices of amyloidogenic 5xFAD mice.** **A)** Silver staining of purified soluble TREM2 species (sTREM2^H157^, sTREM2^222^ and sTREM2^219^) collected from the media of HEK-293T transfected with TREM2 isoforms (TREM2^230^, TREM2^222^ and TREM2^219^) and subjected to electrophoresis in a 4–12% Bis-Tris gel. **B)** Electrophysiology analysis of LTP in C57BL/6J mice in untreated brain slices (aCSF) or incubated with media from HEK cells transfected with an empty plasmid and subjected to the protein purification protocol (Empty). A time-course with the average fESP slopes (% baseline) is shown with an arrow that indicates the time of stimulation (top left panel) accompanied by representative fEPSP trace (bottom panel) and the quantification of the last 10 minutes of fESP slope measurements (% baseline) (top right panel)

**C)**, **D)** and **E)** Electrophysiology analysis of LTP in 5xFAD mice brain slices incubated with soluble TREM2 species at 15 ng/ml. **B)**, **C)** and **D)** correspond to incubations with sTREM2^H157^, sTREM2^222^ and sTREM2^219^, respectively. For each isoform, a time-course with the average fESP slopes (% baseline) is shown with an arrow that indicates the time of stimulation (left panel) accompanied by accompanied by representative fEPSP traces (middle panel) and the quantification of the last 10 minutes of fESP slope (% baseline) (right panel). 4 slices from 2 animals were recorded for all conditions. Statistical analysis was performed by Student t-test. Data are expressed as mean values ± SEM (*p < 0.05 **p<0.01).


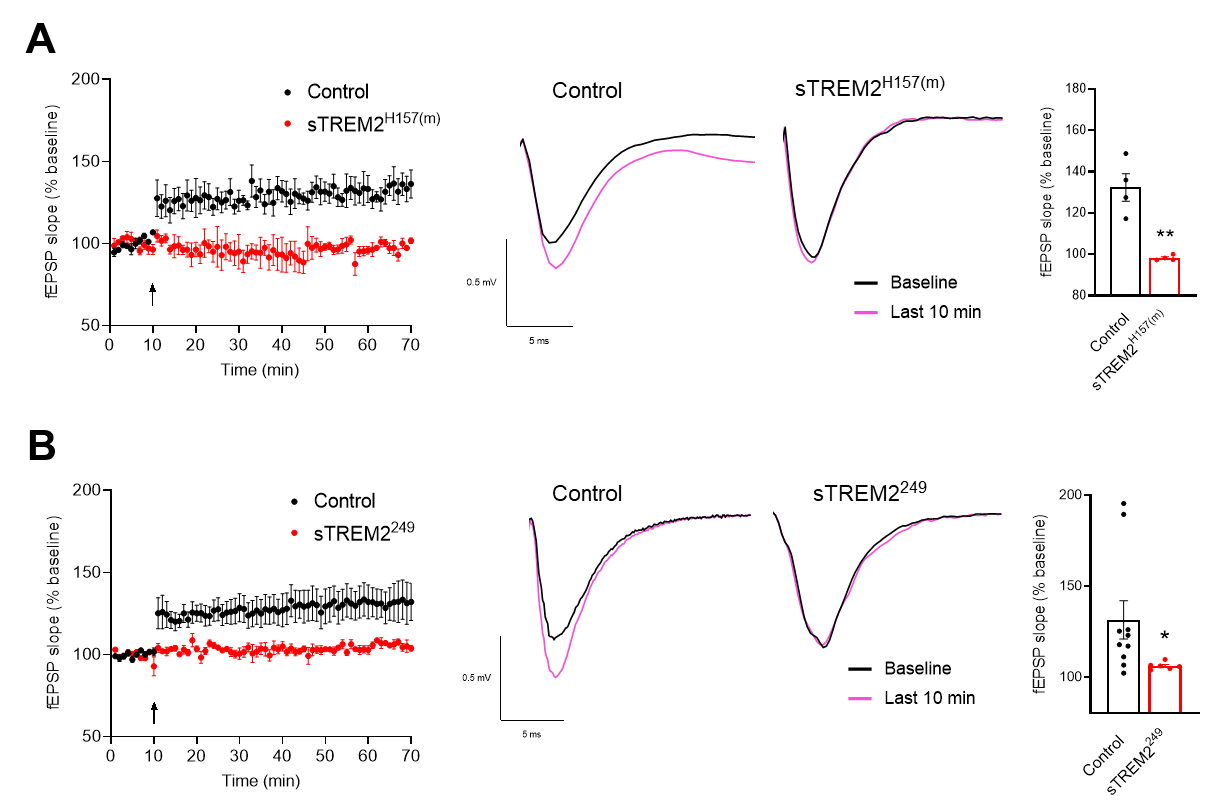


**Figure S4. Murine soluble TREM2 species inhibit long-term potentiation (LTP). A)** and **B)** correspond to incubations of 4-month-old male B6 brain slices with 5 ng/ml of the cleaved sTREM2 species sTREM2^H157(m)^ and the alternative splice isoform sTREM2^249^, respectively. For each isoform, a time-course with the average fESP slopes (% baseline) is shown with an arrow that indicates the time of stimulation (left panel) accompanied by the quantification of the last 10 minutes of fESP slope (% baseline) (right panel). **C)** 4 recordings from 4 animals were recorded for control and from 3 animals for sTREM2^H157(m)^. Statistical analysis was performed by Student t-test. **D)** 10 and 6 slices from 6 animals were recorded for control and sTREM2^249^. Statistical analysis was performed by Mann-Whitney test**.** Data are expressed as mean values ± SEM (*p < 0.05 **p<0.01).

**
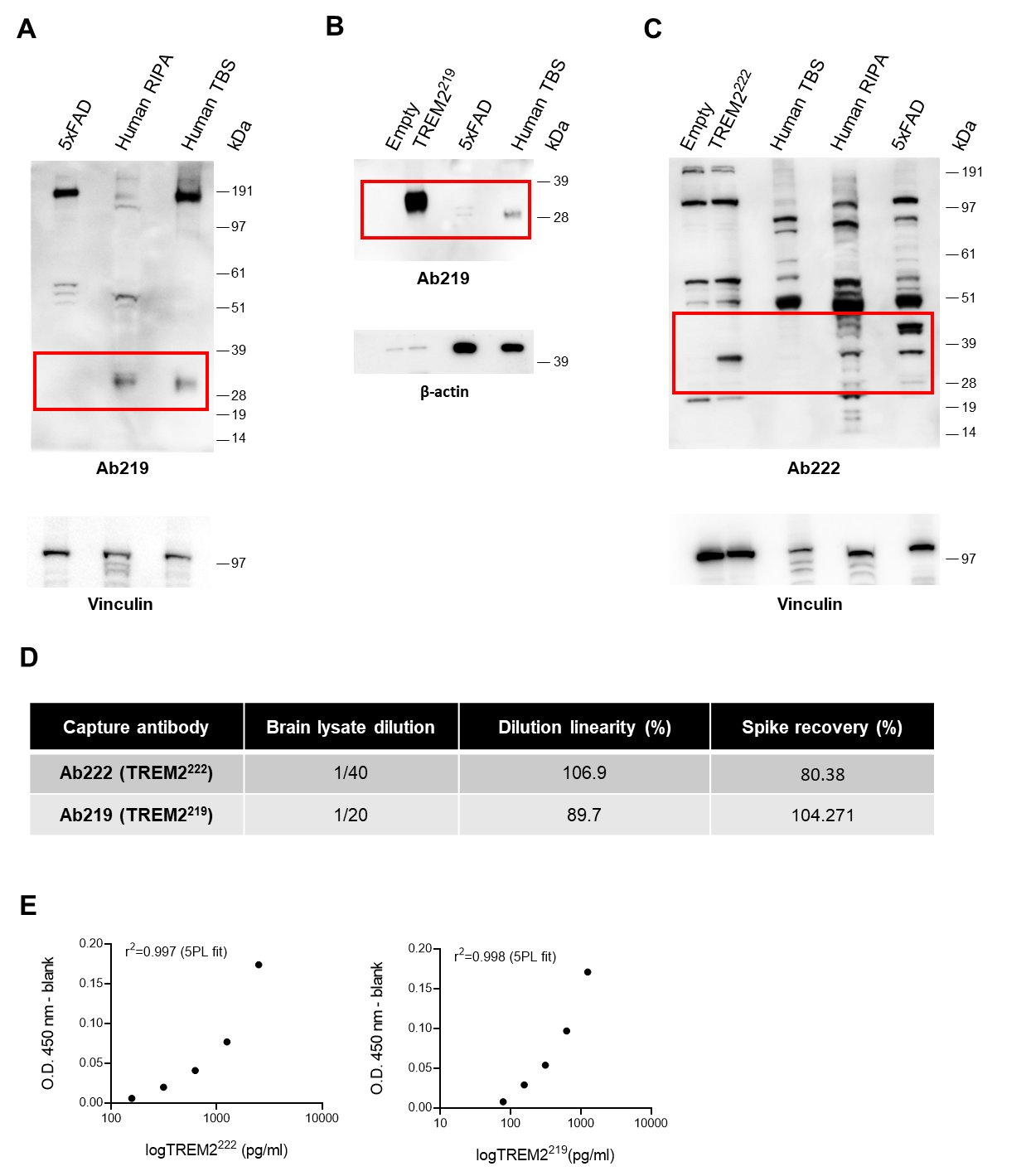
**

**Figure S5. Western blot of TREM2^222^ and TREM2^219^, ELISA dilutions and standard curves. A) and B)** Western blot analysis of TREM2^219^ in 5xFAD cortical extracts, RIPA and TBS-soluble extracts of human brain tissue, and HEK-293T transfected with TREM2^219^ using Ab219 and anti-Vinculin or anti-β-actin as loading controls. **C)** Western blot analysis of TREM2^222^ in HEK-293T transfected with TREM2^222^ TBS and RIPA-soluble extracts of human brain tissue, and 5xFAD cortical extracts using Ab222 and anti-Vinculin as loading control. **D)** Sample dilutions used for the ELISA of TREM2^222^ and TREM2^219^, and respective dilution linearity as well as spike recovery percentage. **E)** Standard curves for detection of TREM2^222^ and TREM2^219^.


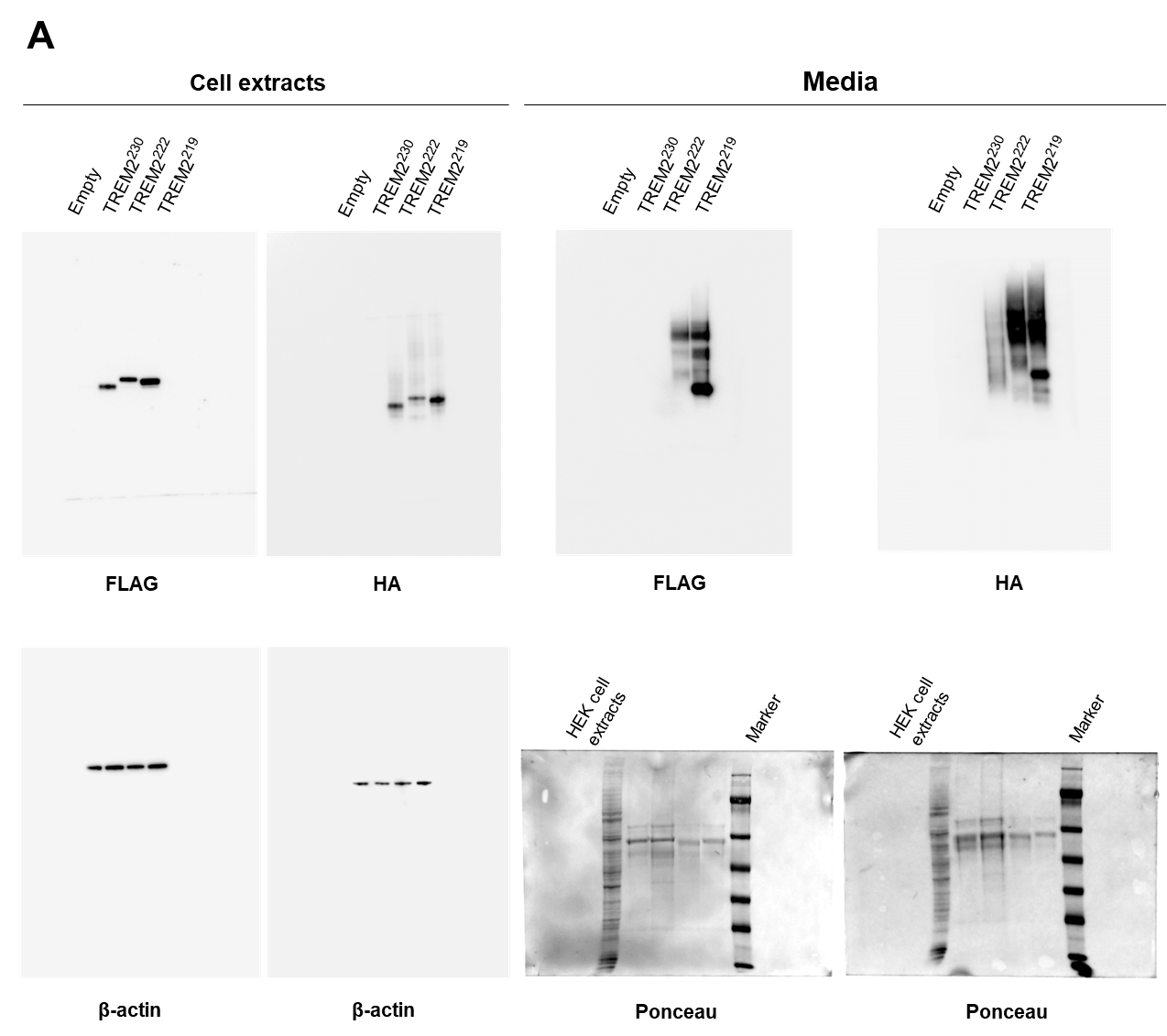


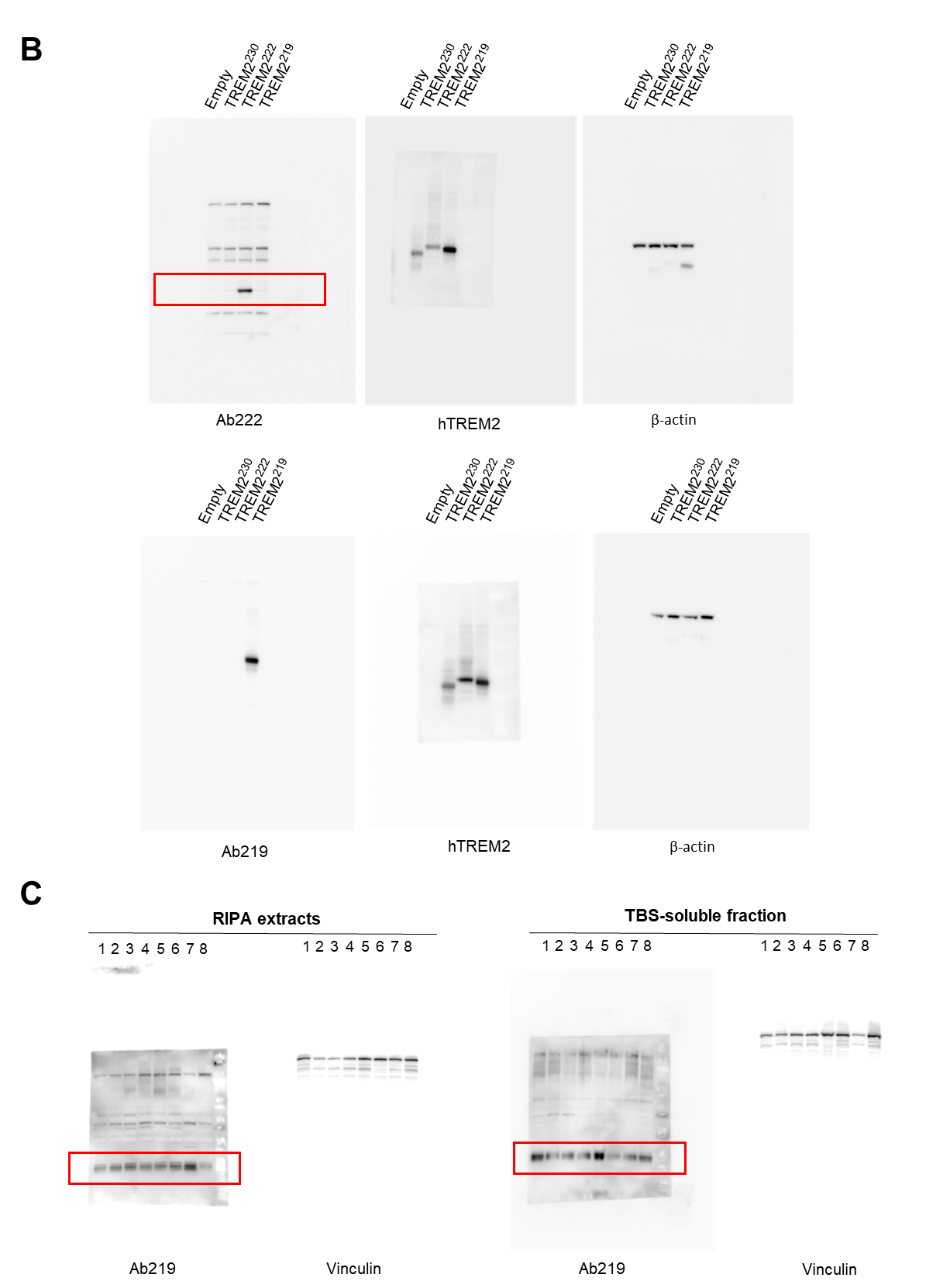


**Figure S6. Uncropped western blots.** Uncropped western blots from figure 2A (A), figure 5B and C (B) and figure 5D (C).
